# Addressing Power Issues in Biologging: An Audio/Inertial Recorder Case Study

**DOI:** 10.3390/s22218196

**Published:** 2022-10-26

**Authors:** Jonathan Miquel, Laurent Latorre, Simon Chamaillé-Jammes

**Affiliations:** 1 LIRMM, University Montpellier, CNRS, 34095 Montpellier, France; 2 CEFE, University Montpellier, CNRS, EPHE, IRD, University Paul Valéry, 34293 Montpellier, France

**Keywords:** bio-logging, low power, audio, inertial data, MEMS sensors

## Abstract

In the past decades, biologging, i.e., the development and deployment of animal-borne loggers, has revolutionized ecology. Despite recent advances, power consumption and battery size however remain central issues and limiting factors, constraining the quantity of data that can be collected and the size of the animals that can be studied. Here, we present strategies to achieve ultra-low power in biologging, using the design of a lightweight audio-inertial logger as an example. It is designed with low-power MEMS sensors, and a firmware based on a small embedded RTOS. Both methodologies for power reduction and experimental results are detailed. With an average power consumption reduced to 5.3 mW, combined with a battery of 1800 mAh, the logger provides 900 h of continuous 8 kHz audio, 50 Hz accelerometer and 10 Hz magnetometer data.

## 1. Introduction

As general awareness of human negative impact on wildlife increases, understanding animals’ responses to short-term disturbances (e.g., activity disruption by people) or long-term changes (e.g., habitat reduction, temperature increase) becomes critical [1]. In recent decades, behavioural ecologists have greatly benefited from the developments in miniaturized electronics: biologging—i.e., the development and deployment of animal-borne loggers—has become a key tool, critical to many studies [2,3,4,5]. Numerous types of biologgers have been developed, to track an animal’s location over time using GPS, monitor its activity using an accelerometer, or record through sound or video collection some information about its environment [6]. Biologging is still a very active field of research with, for instance, for audio-recording, new products appearing regularly [7,8,9,10].

In most species, biologging is strongly constrained by the maximum acceptable size and weight of the logger [3], with the battery usually representing a significant if not a major share of these. Reducing battery size naturally reduces the duration of data acquisition, possibly to the point that data collection becomes useless given the logistical and financial efforts required to deploy loggers, and the fact that capturing an animal biases its behaviour for a while and thus further reduces the actually meaningful data collection temporal window. Battery size reduction could however be achieved with less trade-offs if one is able to compensate for the loss of capacity by a reduced power consumption of the logger. Finding ultra-low power solutions is therefore a clear goal for developments in power-hungry biologging applications, such as audio or video recording.

Achieving ultra-low power is however difficult when using fast-to-develop-on and flexible minicomputers (350 mW announced by the SOLO raspberry-based architecture [11]) or commercial equipment that were designed to have batteries replaced frequently, such as those used and carried by people. Commercial equipment also has the limitation that the logger specifications and behaviour (e.g., recording schedule) can rarely be finely adjusted to the task for which the logger will be used. Overall, more flexible and energy-wise effective solutions can emerge when focusing on dedicated hardware and firmware solutions.

We demonstrate this here in the context of the development of an audio-inertial logger that we previously designed [12,13]. The initial intended application of the device is to record audio and inertial data on large mammals (>20 kg) to better infer their behaviour through biologging. The device would be attached to a collar, and as such, we aimed to have the whole device weigh less than 100 g.

In this article, we describe hardware and firmware choices as well as optimizations made to reduce power consumption. Approaches for energy savings are detailed at several processing stages, from the data acquisition to the mass storage, with embedded audio compression and firmware (including RTOS) tuning in between.

## 2. Hardware Architecture

### 2.1. System Overview

**Figure 1:**
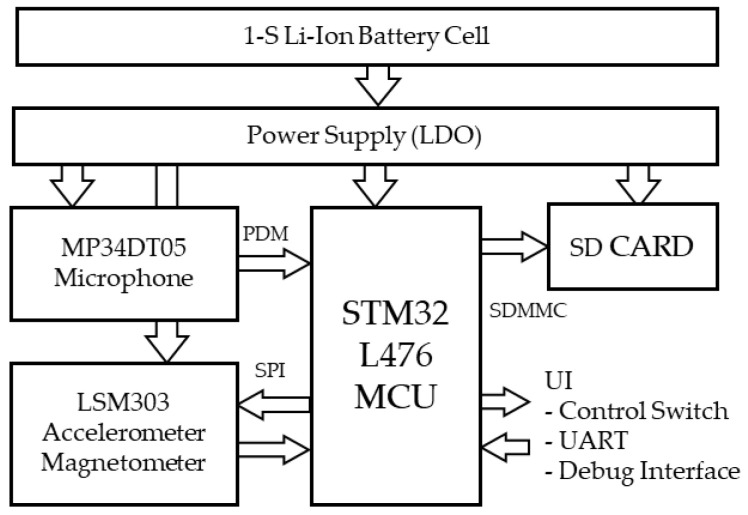
System architecture.

Figure 1 below shows the functional logger hardware architecture. It is simply built around a low-power microcontroller that (i) gathers data from two MEMS devices (microphone and inertial sensing unit) and (ii) stores it onto an SD card. The whole system is battery-powered with a simple low dropout voltage regulator providing a single power domain. The following subsections detail hardware choices that were made for each function.

### 2.2. Sensors

Audio capture requires a microphone. The microphone choice is driven by several concerns; among them are audio performance, output interface, power consumption and integration capability (dimensions, assembly). Because a microphone must be somewhat exposed to the environment to “hear” incoming sounds, mechanical robustness and water resistance are also to be considered. Finally, cost (including integration) is an obvious criterion. Regarding the output interface, microphones deliver audio data either as an analog signal or by means of a digital interface. The MEMS microphone market offers a wide range of equivalent solutions in terms of performance, cost, integration, and robustness. The most important design option concerns the signal output format, either analog or digital, with marked difference in terms of intrinsic power consumption. We investigated both.

**Figure 2:**
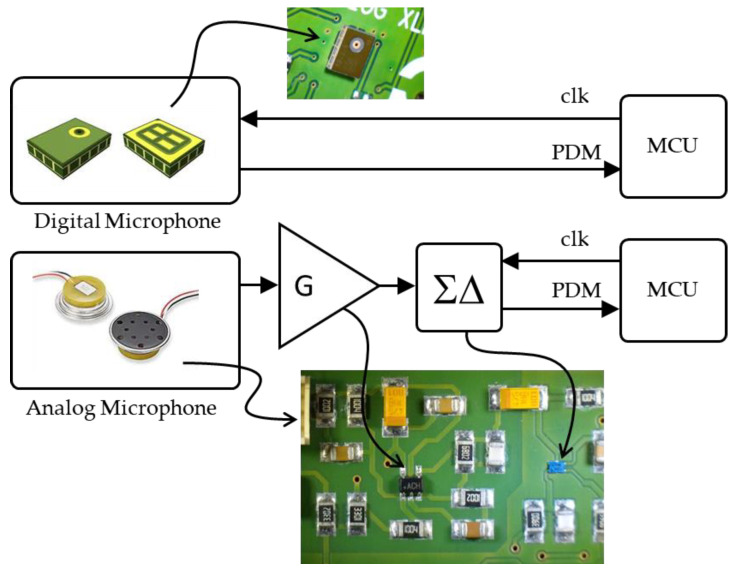
Microphone options. (**Top**): digital, (**Bottom**): analog.

Analog microphones offer lower intrinsic power consumption but require extra on-board analog signal processing such as amplification and anti-alias filtering. These introduce additional energy and integration costs. Conversion into the digital domain must then be performed using either (i) MCU-available ADC or (ii) an external converter. Because of the limited resolution of embedded ADCs (typically 10 to 12 bits), the latter solution is preferred. The requirement for anti-alias filtering can be circumvented using a ΣΔ modulator that produces effective noise shaping. A validated solution for analog microphones was introduced in [12] and is shown in the bottom part of Figure 2. It is based on a waterproof MR28406 microphone from Knowles, a discrete FAN3850A ΣΔ modulator from Fairchild and additional 18 dB on-board amplifier. We measured the supply current of the sole audio chain (microphone + amplifier + ΣΔ modulator) under the direct application of a nominal 3 V supply voltage from a laboratory DC source, and with a 2 MHz clock provided to the ΣΔ modulator. We observed a total power consumption of 3 mW, close to what we can achieve with a fully integrated digital microphone. Note that the assembly of an analog microphone, an amplifier stage and a ΣΔ modulator is nothing else than building a digital microphone on board. The only advantage of the analog choice for the microphone is the availability of hardened products that better resist harsh environments. It comes at the cost of integrating more components on board, a higher footprint, and no benefit on power consumption.

For these reasons, the actual logger integrates a digital MEMS microphone (MP34DT05 from STMicro) as shown in the upper part of Figure 2. It offers a cost and footprint effective solution with a 2 mW power consumption (as measured under the same conditions as for the analog approach: 3 V, 2 MHz). Audio is delivered as Pulse Density Modulation stream (PDM), which corresponds to the output of a ΣΔ modulator. Audio quality will be discussed later, in the results section. Such microphones are not designed for severe environments and must be protected from humidity at the logger package level.

Movement capture requires an inertial sensor unit: 9-DOF inertial measurement units commonly cover three-dimensional accelerations, rotation speeds and orientation with respect to the Earth’s magnetic field. Due to their inherently higher power consumption, gyroscopes have not been considered for this application. A 6-DOF device was retained (LSM303 from the STMicro E-Compass product range) providing three-axis acceleration and three-axis magnetic field orientation.

### 2.3. Data Storage

Regarding data storage, the main characteristics besides power consumption are the storage capacity, the transfer bandwidth (both for writing and for recovering data), the integration capability, and the cost. In the context of our application, the amount of data to store ranges from a few kB/s to a few tens of kB/s, which is low regarding the existing physical storage options, and therefore is not a limiting factor. However, for a 1000 h campaign, collected data reach several tens of Gigabytes. We therefore need high storage capacity, reasonable writing times, and a fast-reading approach to recover a large amount of data. The obvious option is a removable SD card. It provides a cost-effective solution and easy data recovery by directly interfacing with a computer (assuming data writing uses a standard file system layer).

### 2.4. MCU

The market is not short in low-power microcontrollers that could fulfil our logger requirements. What makes a difference is the ability to interface with on-board sensors and mass storage devices. An interesting feature available on few STM32 products is a peripheral called DFSDM (Digital Filter for Sigma Delta Modulators) which basically implements clock generation and a decimation filter suitable with PDM digital microphones. Together with a DMA (Direct Memory Access) controller, one can foresee the buffering of audio samples (from microphone into memory) without any CPU activity, which is a good start in an energy-saving context. Based on this idea, our final choice is a STM32L476 device from the low-power STM32 range. Based on an ARM Cortex-M4 CPU, this microcontroller might seem overstated at first glance. However, native SD interface (Secure Digital and Multi-Media Card) is only available on high-end MCUs. Finally, as it will be later discussed, having some on-chip processing power can be interesting, even in the context of power saving.

## 3. Data Flow

What we call data flow here are the steps required to transform the physical information (measurand) to its final digitized form on a storage drive.

- At the front end, an acquisition is performed by a sensor (transceiver) that transforms the physical input into raw digital data.
- At the back end, storage saves data into the non-volatile memory.
- In between, the data are processed to accommodate acquisition and storage formatting and to address global performance.

Addressing power consumption concerns every stage of this data flow. The following subsections provide details regarding the design of each one.

### 3.1. Data Acquisition

#### 3.1.1. Microphone

When powered, the MP34DT05 microphone continuously transmits audio data in the form of a PDM signal, which frequency corresponds to the frequency of the provided clock. This signal is converted into audio samples by the DFSDM hardware, which in turn requests DMA transfer each time a new sample is available. The process of buffering audio samples into MCU memory is therefore fully performed by a dedicated hardware and does not involve CPU. The DFSDM peripheral is also in charge of generating the clock driving the microphone internal ΣΔ modulator.

The PDM stream is filtered by the DFSDM peripheral and converted into a 24 bit audio sample using the following z-domain transfer function:

(1) $$H\left(z\right)={\left(\frac{1-z^{-FOSR}}{1-z^{-1}}\right)}^{X}$$

where *FOSR* is the oversampling ratio and *X* the sinc filter order. Setting *X* = 2 for instance produces the corresponding function for a second order filter:

(2) $$Y_{n}=2Y_{n-1}-Y_{n-2}+X_{n}-2X_{n-FOSR}+X_{n-2FOSR}$$

where *Y_n_* represents the *n*th audio sample (output) and *X_n_* the *n*th input bit.

Both *FOSR* and *X* are parameters that can be set by software to adjust the filter response according to microphone characteristics and targeted audio quality. The filter output can be further averaged by an additional summing stage (integrator) that reduces throughput while increasing the dynamic range. The corresponding sample rate *SR* is given by:

(3) $$SR=\frac{f_{clk}}{FOSR\times IOSR}$$

where *f_clk_* is the ΣΔ modulator clock frequency, and *IOSR* is the number of samples to sum-up before delivery. From there, audio bandwidth can be deduced with the Shannon criterion:

(4) BW=SR/2

Finally, the filter output full scale can be adjusted for a given *FOSR* by adjusting the filter order *X*:

(5) $$FS=\pm FOSR^{X}\times IOSR$$

This allows for tuning the microphone “digital” sensitivity independently from the bandwidth.

A higher sampling rate provides better audio quality. In this study, two audio configurations have been investigated to target two different mission profiles. Referring to sample rate, the so-called “32 kHz” version focuses on the audio quality, covering most of the human ear audio bandwidth. The so-called “8 kHz” version is a downgrade in audio quality. With a reduction by four of the data amounts to process and store, this version is expected to provide a better autonomy/weight ratio. It is aimed for applications where the requirements in audio quality are not critical, yet are appropriate for machine-learning-assisted classification.

Parameters for both alternatives are summarized in Table 1. Note that microphone clock frequency of 2 MHz is set according to the datasheet.

#### 3.1.2. Inertial Measurement Unit

The LSM303 inertial measurement unit is made of two independent devices (accelerometer and magnetometer) packaged together and sharing the same SPI bus (with distinct select lines) for data transfer to and from the MCU. Two individual interrupt lines are available for signalling when new data are available. The SPI transaction is then initiated by software. The CPU is therefore required for a short time, for every new three-axis data sample.

Output data rates are set separately for each sensor: 50 Hz for the accelerometer and 10 Hz for the magnetometer. They rely on an internal RC clock that is neither precise nor stable in frequency. Moreover, as there is no hardware synchronization between inertial data and audio data, a software alignment approach has been implemented.

### 3.2. Data Storage

Data are written onto an SD card using the standard File Allocation Table (FAT) file system. Audio is directly written as standard WAV files, allowing the end user to read and play recorded audio from basically any computer/OS. Inertial data are written as binary files that require some post-processing (e.g., Matlab^®^, Python, R, …) before they can be exploited.

Hardware-wise, an SD card is driven by the MCU SDMMC peripheral that handles native standard 4 bit SD transactions. Making use of the available DMA, the time-consuming transport of large data chunks, from the MCU memory, over the SDMMC and to the SD card, is fully operated by dedicated hardware, leaving the CPU free for other tasks, including sleeping. The CPU is only required for a short time at the beginning and at the end of the transaction.

However, SD card operations are energy-hungry processes that deserve some focus. In a separate study using the same hardware, we developed a dedicated firmware that repeatedly performs dummy SD card writings, while varying the length of the data chunk. The transient supply current is captured using a Keysight CX1102A current probe connected to the main supply and a CX3324A waveform analyser. Note that no sensors are being powered during this measure, therefore observed energy only accounts for the linear 3 V LDO stage, the MCU (including CPU, DMA and SDMMC) and the SD card. The supply is provided by a laboratory DC source mimicking a fully charged 1S Li-Ion battery (4.2 V). An MCU output pin is toggled to signal the beginning and the end of the data transfer so that energy can be further calculated by summing transient current over the writing time window.

**Figure 3:**
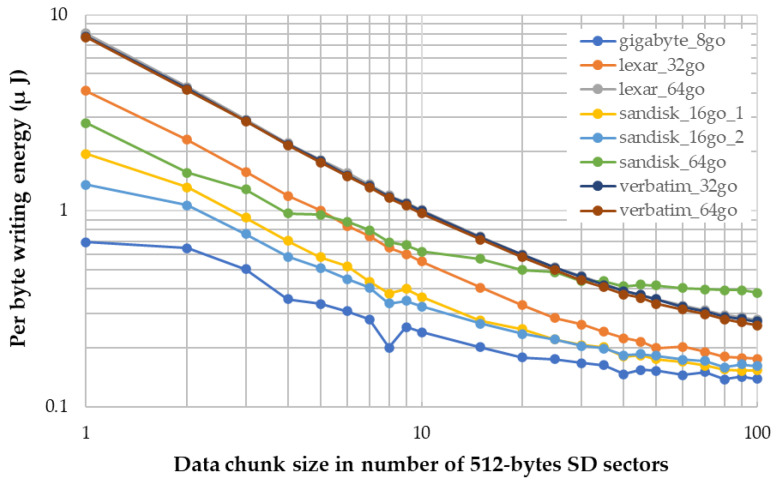
Measure of the “per byte” energy required for SD card writing operation.

Figure 3 shows the “per byte” energy requirement (log-log scale), measured on a selection of SD card candidates. The *X*-axis represents the size of the data chunk to be written in one go, expressed as an entire number of SD card sectors of 512 bytes. Given that variability between writing cycles on the same card is important, Y-value energies are obtained by averaging the results over 100 writing cycles with the same parameters (SD card model and data chunk size).

The obtained results demonstrate that it is always more efficient to write a larger chunk of data in one go. This can be explained by the constant power overhead that is required to initiate and to stop data transfer. The SD card manufacturer does not provide power consumption information. As expected, notable variations occur across different SD card capacities and brands, but the common pattern is a logarithm decrease in the per byte energy along with the data size. Higher-capacity cards (64 Gb) tend to require more energy than their lower-capacity counterparts. The graph also shows that beyond about 40 sectors (i.e., writing chunk > 20 kB), the per-byte energy seems close to reaching a floor below 0.2 µJ with lower-capacity cards.

### 3.3. Data Processing

#### 3.3.1. Inline Audio Compression

With a *FOSR* = 256 and a 2 MHz clock, the microphone generates 32 bit samples at 7812 Hz. Audio samples are shrunk (roundoff) to 16 bit integers, bringing the data rate of the microphone to 15,624 bytes per second. With such throughput, filling a buffer corresponding to 15 SD sectors (7680 bytes) takes a mere 491.55 ms. Although this process requires very little CPU, it generates considerable SD activity.

**Figure 4:**
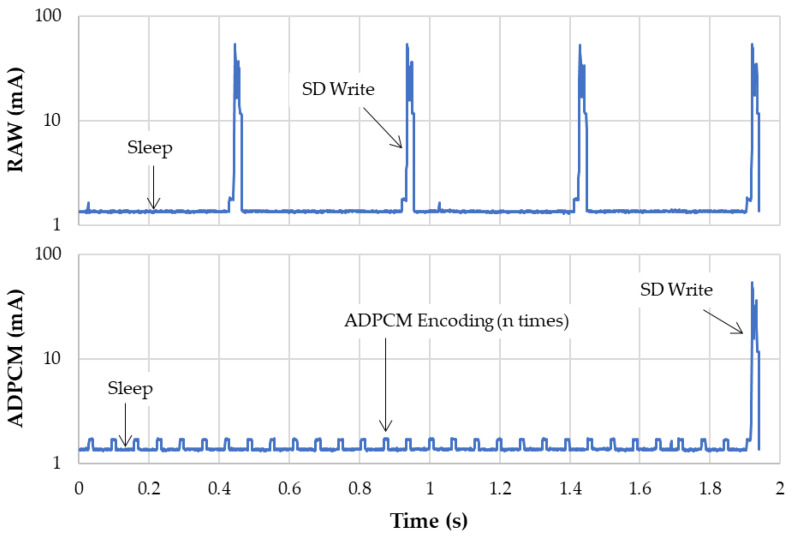
Comparison of transient currents during audio recording without (**top**) and with (**bottom**) embedded ADPCM compression.

Two approaches were investigated to manage this amount of data. The first one is a straightforward saving of raw audio 16 bit samples, keeping the CPU in a low-power mode as much as possible. The second approach is to implement a software data compression before saving, thus increasing the CPU usage for an expected benefit at storage level. To achieve this, the compression algorithm must be simple enough for fast execution, while still providing an effective compression ratio. ADPCM ticks both properties. It is a light-weight algorithm that encodes a 4 bit difference between two consecutive samples achieving a 4:1 compression ratio at low CPU cost. To prevent drift over time, the WAV-ADPCM format imposes to process input data in small chunks of 505 samples, thus creating ADPCM blocks of 256 bytes made of 504 compressed samples (252 bytes) and a 4-byte header containing one plain 16 bit sample to keep the common mode under control. Figure 4 compares the current consumption of the logger during a ~2 s sequence of audio recording without (top) and with (bottom) the embedded ADPCM compression. Reported currents are measured at main 4.2 V supply, using the same equipment as for the previous SD card study and thus include the whole audio system power consumption including the 3 V onboard voltage regulator. To make the comparison fair, the same amount of data is written onto the SD card every time it is required, placing both approaches at the same arbitrary per-byte writing efficiency, corresponding to chunks of 15 SD sectors. Without compression, the microcontroller is left in a sleeping state between SD card operations. At 7812 Hz, these operations occur every (15 × 512)/(7812 × 2) = 0.49 s. When compression is engaged, the CPU is woken up every 505/7812 = 64.6 ms, but the period between the SD card writes is now four times greater (1.94 s).

Table 2 reports the energy required by the two approaches during the same 2 s window frame. As a result, inline audio ADPCM compression not only saves 75% mass storage space but also saves about 30% of power consumption. Because ADPCM is a destructive algorithm, this obviously impairs audio quality. This matter is addressed later in the result section. For now, and for the sake of autonomy, compression is retained as a de facto implementation in the firmware.

#### 3.3.2. Buffering Strategy

**Figure 5:**
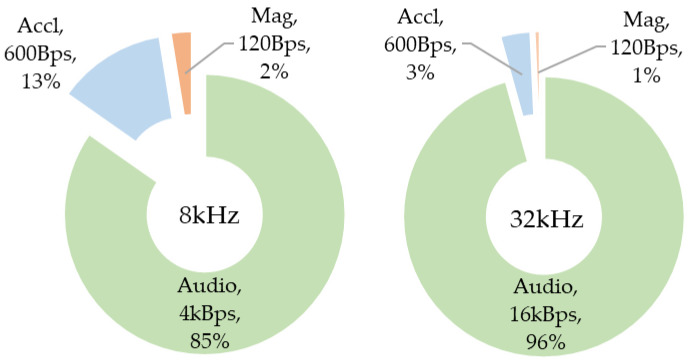
Data source contribution to mass storage (given in byte/s data rate).

Knowing that the audio compression divides by a factor 1:4, the audio data rate on the SD card, we can calculate each sensor contribution in the SD card activity and deduce an optimal buffering strategy regarding the “per byte” writing efficiency. Figure 5 below summarizes the amount of data to store. It shows that even in 8 kHz configuration of the logger data amount is mostly defined by the compressed audio size, making it the determining point in SD card writing efficiency.

**Figure 6:**
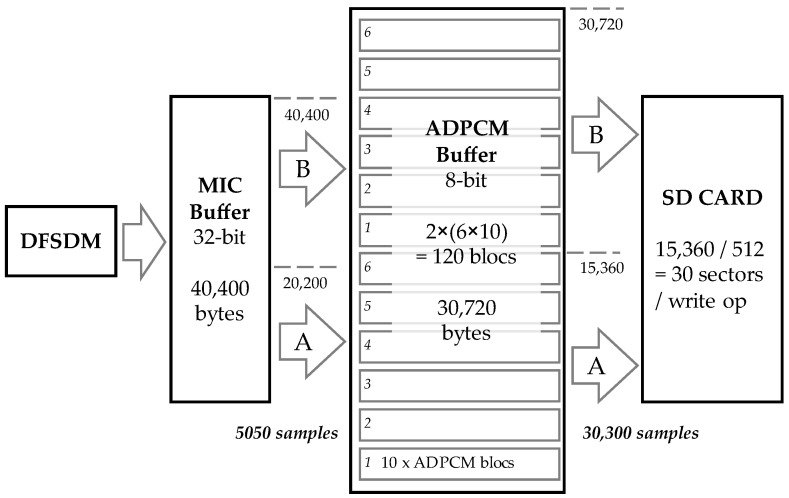
Audio data buffering scheme.

Figure 3 clearly shows that the per byte writing efficiency improves with larger data chunks. It is therefore obvious that some level of buffering scheme must be implemented in the MCU before storage. Regarding audio data and considering ADPCM compression, we need two buffering layers:

- First layer: A raw audio data buffer (32 bit samples) that is automatically filled by audio samples coming from the microphone-DFSDM-DMA hardware stream. It is labelled MIC buffer in Figure 6.
- Second layer: An ADPCM-compressed audio data buffer (4 bit samples). This buffer is filled by software every time the CPU performs ADPCM compression on raw audio samples. It is labelled ADPCM buffer in Figure 6.
- A simpler buffer scheme applies to inertial data with two additional buffers:
- An accelerometer data buffer (3 × 16 bit samples). This buffer is filled by the CPU every time an accelerometer interruption occurs.
- A magnetometer data buffer (3 × 16 bit samples). This buffer is filled by the CPU every time a magnetometer interruption occurs.

Let us focus on audio data first, which represents the larger data source. If we only consider memory saving, one should therefore call for data compression every 505 audio samples (i.e., one single ADPCM bloc) and retain audio data mostly in its compressed 4 bit form. However, doing so would call the CPU for a compression task every ≈ 60 ms or every ≈ 15 ms for the 8 and 32 kHz versions, respectively, making low-power strategies difficult to deploy.

For this reason, we sized the first buffering layer, receiving raw samples from the DFSDM peripheral so that 10 ADPCM blocks can be computed every time the CPU is called (i.e., 5050 32 bit audio samples). For this layer being automatically filled by DMA, a synchronization mechanism must be implemented to avoid read and write collisions. This is performed by means of a feature of the DMA controller that can generate interruptions when either half or a complete transfer is achieved. For that reason, the first layer is doubled in size so that 5050 samples are stored in the lower half “A”, and another 5050 samples are stored in the upper half “B”. When DMA signals that a half buffer has been filled (either A or B), software can safely read data from that half buffer, while DMA starts filling the other one, the process being circular. A continuous stream of incoming data can be seamlessly processed this way, assuming that the reading and processing is faster than the filling. The first layer is therefore sized for 10,100 samples, corresponding to 40,400 bytes.

The second buffer layer (ADPCM buffer in Figure 6) is sized to retain 60 ADPCM blocks so that the SD card writing is performed in chunks of 256 × 60/512 = 30 sectors. Referring to Figure 3, this places us in a very good per-byte writing efficiency, close enough to the supposed floor. The same A/B half-buffer strategy as for first layer is implemented in the software here. This is performed to enforce the second layer robustness, making it able to cope with huge SD card writing time variability (from ≈40 ms most of the time up to ≈1 s in casual worst cases). The second layer size is then 2 × 60 × 256 = 30,720 bytes.

The first layer calls for ADPCM compression when 20,200 bytes have been written in the MIC buffer (At 8 kHz audio, it occurs every 631 ms). The result of the compression is 10 ADPCM blocs (15,360 bytes) which are stored in the ADPCM Buffer. When this process has been repeated six times, the ADPCM buffer is half full, thus triggering an SD card writing of 15,360 bytes corresponding to 30 full sectors on the SD card (this occurs every 3.7875 s at 8 kHz audio). At 32 kHz, the amount of data generated is multiplied by a factor four and so are both the processing (158 ms) and SD card writing (0.947 s) frequencies.

**Figure 7:**
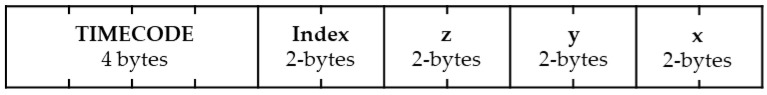
Structure of the 12-byte array representing a timestamped 3D inertial sample.

Regarding inertial data, rates can be configured in the firmware. At this moment, we have been working with fixed rates of 50 Hz for the accelerometer and 10 Hz for the magnetometer. These rates are under the control of the sensors themselves, which do not provide stable and precise time bases. Since it is important for the subsequent data analysis to perfectly align the audio and inertial data, a 32 bit time code is embedded with each single inertial sample. An actual sample record structure is shown in Figure 7. The timecode is based on a 32 bit system counter with typical resolution of 1 ms rolling over about every 50 days, which is more than comfortable for easy data alignment. Note that the audio is timestamped as well using the same counter. However, since the audio rate is precisely controlled by an external RTC crystal oscillator, audio timestamping is only performed once per hour with the timecode inserted into WAV file header.

**Figure 8:**
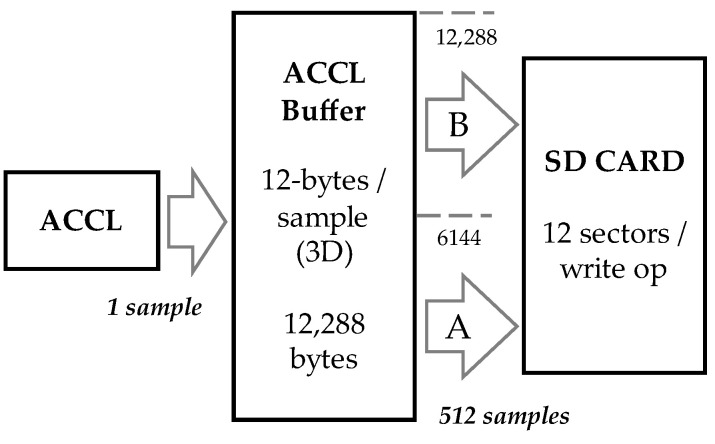
Accelerometer data buffering scheme. The same applies to magnetometer data.

Inertial data are buffered into arbitrarily sized 6 kB buffers, doubled to 12 kB as for audio data to implement an A/B approach as shown in Figure 8. At 50 Hz, accelerometer data fill a buffer every 6144/(12 × 50) = 10.24 s, triggering an SD write operation on 6144/512 = 12 sectors. Magnetometer data are written on the SD card every 6144/(12 × 10) = 51.2 s.

Table 3 summarizes RAM usage based on a total of 128 kB available (STM32L476RE). Up to 73% is dedicated to data buffering to optimize mass storage operations both in terms of power efficiency and robustness. The rest of the memory is used by firmware, including RTOS heap that supplies memory for kernel objects and tasks stacks, and statically allocated global variables.

## 4. Firmware Tuning

### 4.1. Software Architecture

Because data delivery among the three sensors is intrinsically asynchronous, and because SD card writing times are not deterministic, a bare-metal sequential process is not the best approach for firmware development. The software architecture therefore relies on the use of a real-time OS (FreeRTOS) that provides a better CPU resource sharing approach.

**Figure 9:**
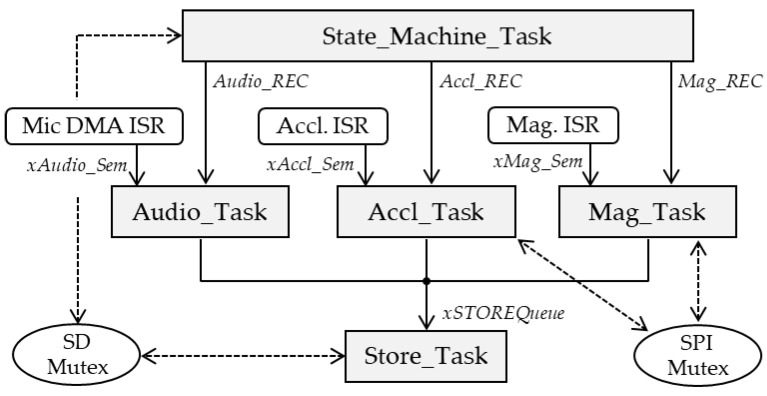
Firmware RTOS architecture.

Figure 9 shows the event-based software architecture used in the logger. At the lower level, three tasks handle data collection. The *Audio_Task* waits for DMA interrupts and subsequent *xAudio_Sem* semaphore to process the MIC buffer and store compressed samples into the ADPCM buffer. When the ADPCM buffer is full, the *Audio_Task* pushes a message into the *xSTORE_Queue* FIFO. Both *Accl_Task* and *Mag_Task* work on the same principle, waiting for sensor interruptions and corresponding *xAccl_Sem*, *xMag_Sem* semaphores and then pushing a message into the same *xSTORE_Queue* queue. The *Store_Task* is activated upon message availability in the *xSTORE_Queue* FIFO. Depending on message origin, this task records data onto the SD card, using separate files for the audio, accelerometer, and magnetometer data. Because the accelerometer and the magnetometer share the same package and SPI bus, a mutex (*SPI_Mutex*) is used as resource protection to avoid two tasks trying to access the bus at the same time. At the top of the software hierarchy, the *State_Machine_Task* oversees the logger sequencing, SD card file management, and user interface for initialization (time and date settings) and recording start/stop. Because both *State_Machine_Task* and *Store_Task* may access the SD resource, it is protected by means of a mutex. Finally, the user can program recording windows with a subset of data to be recorded. For that purpose, the *State_Machine_Task* can toggle (on/off) each data-collecting task by means of a special kind of non-resetting semaphores (*Audio_REC, Accl_REC, Mag_REC*) working as blocking flags (event group).

### 4.2. Dynamic Power Management Policy

**Figure 10:**
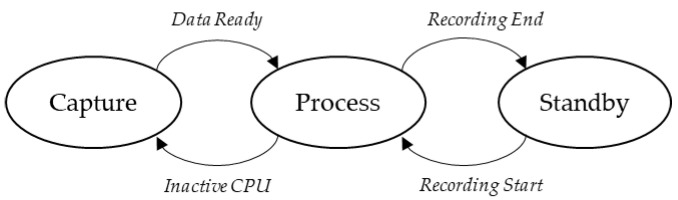
Dynamic power management policy.

The idea behind a Dynamic Power Management (DPM) policy is to clearly identify runtime device states and then only supply what is strictly required in each of these states [14]. In our case, three states can be distinguished, which are illustrated in Figure 10.

- A “Capture” state during which (i) audio samples are collected and put into the MIC buffer with no CPU load, and (ii) the inertial sensor is performing its measure.
- A “Process” state during which the CPU is called for either (i) ADPCM compression, or (ii) inertial data reading and subsequent casual (iii) SD card writings.
- A “Standby” state that represents the deepest low-power state. It is the default state before and after a scheduled recording is performed. During this state, it is assumed that only minimal hardware resources are required to keep track of time and date (MCU’s Real-Time Clock (RTC)).

Let us put the Standby state apart, as it will be addressed later. Interesting states are “Capture” and “Process” because the device continuously commutes from one state to the other during recording, with CPU switching between on and off states accordingly. Although it is a trivial use case when coding bare-metal, the use of an RTOS makes the situation substantially more complicated. The RTOS scheduler is called at regular time intervals by a hardware interruption (tick) that compromises power efficiency, making the CPU impossible to keep sleeping over a long period.

Still, when no operational functions require CPU attention, the RTOS enters a default “Idle” task. This event can be detected, and then two tactics for addressing power consumption can be setup [15]:

- The “Idle Hook” approach: The hook is a function called by the OS scheduler whenever it enters the idle task. The role of this hook is simply to disable unused peripherals and set the CPU in a sleep state. Exit from this state occurs upon any event (either a sensor interrupt or an OS event, including ticks). This method introduces very little overhead to the code execution. However, the CPU is still activated for a short time every OS tick so that it can be combined with an increase in tick periods.
- The “Tickless Idle” approach: It consists in preventing OS ticks to occur when there is no CPU load, which in practice means suspending execution of the OS scheduler. Doing so requires careful setup of extra mechanisms that (i) keep track of time during the tickless period instead of the OS and (ii) allow for exiting the idle state when an event requiring the CPU occurs. Expected events can be a sensor interruption or the end of a programmed delay. This approach introduces small execution overhead for entering and exiting the tickless mode.

**Figure 11:**
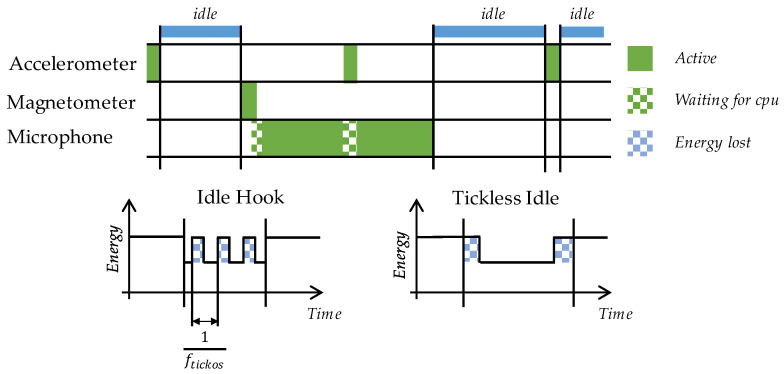
Low power strategies when using RTOS.

Each tactic therefore has its pros and cons. Figure 11 illustrates where energy is lost using either the Idle Hook or the Tickless Idle approach. Note that the Idle states are used to put the microcontroller into one of its low-power modes. There are several levels of energy savings depending on the amount of hardware resource that is turned off, while turning off can be applied to the clock, power supply or both. In our case, the MCU peripherals and RAM are always active because they are involved in the audio data capture. This limits possible low power mode to simply stop the unused peripherals and CPU clocks.

Table 4 presents the average consumption during the Capture (Idle) and Process states. Under normal conditions, with a 4 MHz CPU clock, we observe a 1.32 mA current consumption when the MCU is in low power mode, and 1.65 mA when the CPU is performing tasks. That is a 20% difference, making the implementation of a low-power strategy worthy.

**Figure 12:**
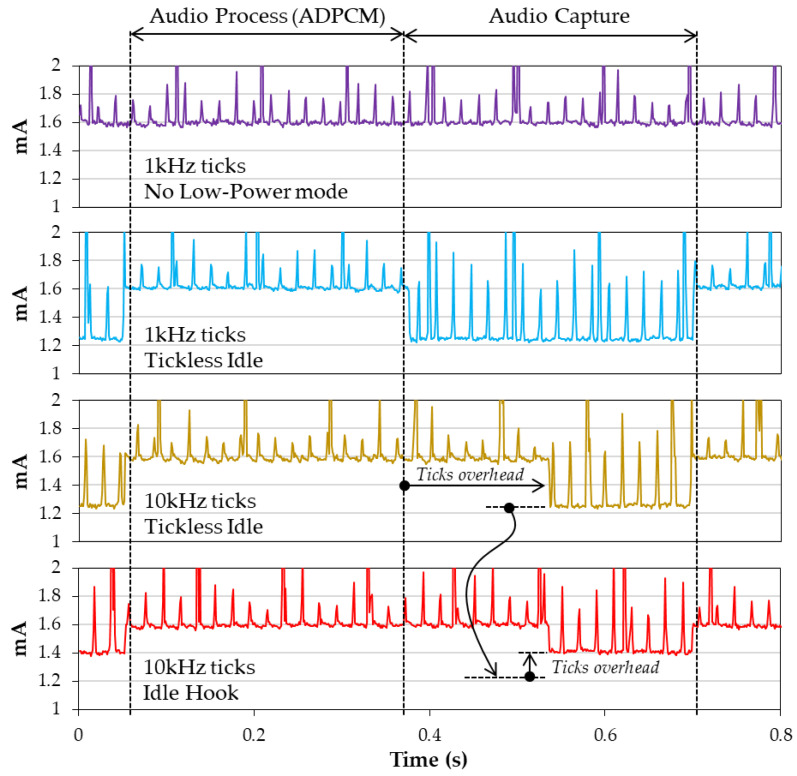
RTOS low-power strategies.

Effects of tick rate are not visible for lower frequencies (≤1 kHz). The reason is that tick density is in practice very low compared to the CPU execution cycles and that the power loss due to regular wakeups is negligible. That is the reason why both Idle Hook and Tickless Idle exhibit about the same performance at tick rates of 1 kHz and below. However, if the tick rate is raised to 10 kHz, the Capture state managed with the Idle Hook strategy shows higher average power consumption (1.47 mA), while this consumption remains unaffected using the Tickless Idle option, for obvious reasons (ticks are stopped in this case). In addition, pushing the tick rate too high is never a good idea because it increases the OS self-contribution in the total CPU load, making in practice the CPU less responsive to application tasks. This phenomenon is well illustrated in Figure 12.

From these results, it appears that keeping a 1 kHz tick rate, associated with a Tickless Idle low-power state management, provides a good trade-off between energy consumption, OS responsiveness and timing robustness.

### 4.3. Build Optimizations

Previous attempts to improve power efficiency essentially focused on the vertical axis of the problem (i.e., the amount of required supply current for each state). Let us now examine the horizontal axis (time). Can we make the process state shorter with respect to the capture state? Or in other words, improve the activity duty-cycle ratio? During the process state, the most demanding task is ADPCM encoding (remember that SD card operations have been left apart). The algorithm itself is simple and provides no opportunity for a more efficient code. However, the compiler optimization level set at build time has, as expected, a direct effect on the execution performance. Because it makes debugging less easy, these optimizations are not engaged is early development phases, nor necessarily on the whole firmware code. Here, let us examine the effect of compiler optimization applied on the sole APCPM algorithm function code.

Table 5 presents CPU usage depending on compiler optimization level. We note that enabling the first level of optimization (−O1) reduces ADPCM compression execution time almost by a factor three. Then, no further noticeable improvement is observed with higher optimization levels. Explaining the faster code execution under compiler optimization is easily performed by analysing the generated assembly code. A simple variable assignment (=operator in C) leads to three atomic operations when no optimization is engaged: (i) loading data from RAM, (ii) performing assignment, and (iii) saving result back in RAM. With −O1 optimization and beyond, loading from and saving to RAM is only performed outside loops. Within loops, variables are replaced by CPU registers, then acting as a cache and saving unnecessary multiple access to the RAM memory, effectively producing a 1:3 performance improvement in code execution.

**Figure 13:**
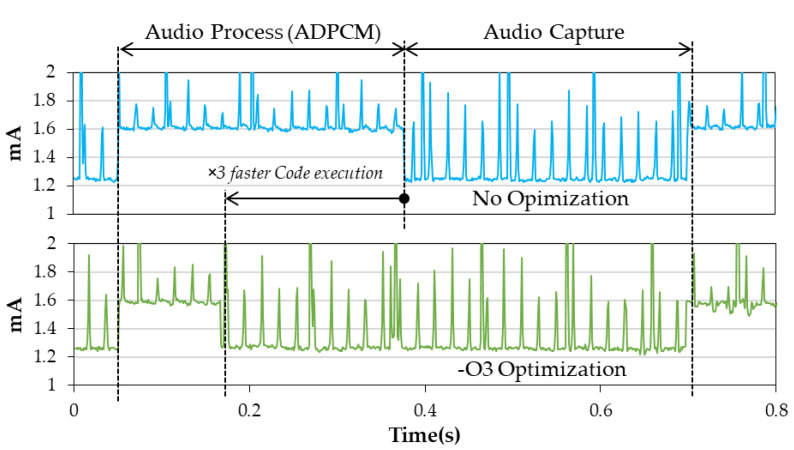
Effect of compiler optimization applied to the ADPCM algorithm on power consumption (1 KHz Tickless Idle mode).

Figure 13 illustrates the effect of ADPCM optimization on transient currents. Having a ×3 faster compression algorithm produces an additional 8% gain in the logger power consumption, which is not negligible.

### 4.4. CPU and Peripherals Clock Frequency

The MCU’s internal clock frequencies have a strong impact on the global power consumption and should be selected carefully, as low as possible according to the application requirements. Two factors need to be considered here: peripheral requirements and CPU load. In our case, the major constraint comes from the digital microphone that requires a fast 2 MHz clock for its ΣΔ modulator. Because of the internal MCU clock tree architecture, the minimal CPU clock frequency is then twice that of DFSDM peripheral, resulting in a 4 MHz CPU clock frequency. Note that this is low in the context of modern MCUs and way below the maximum frequency of 80 MHz this CPU can accept. Furthermore, running the MCU at low speed allows for internal power regulators to work in a more efficient mode.

Regarding CPU load, and after compiler optimization is engaged, we confirmed that the 4 MHz clock frequency provides enough computing ability for both the 8 and 32 kHz audio data rates.

### 4.5. Standby Mode and Recorder Scheduler

The logger relies on MCU’s internal RTC (Real-Time Clock) to split into files, label and timestamp recorded data. This RTC must be set to the actual time and date before the logger mission starts. This is performed in the lab and for the time between RTC setup and recording, and the logger is put in a standby mode where only the RTC domain remains powered with an ultra-low current consumption of 35 µA. For field experiments that do not require a 24/24, 7/7 recording, it is possible to start and shutdown the recording process during predetermined time slots. These time slots are user defined and are written (using an ad hoc web-based application or a R script) in a formatted text file to be put on the logger SD card before powering on. The scheduler then puts the logger back in standby mode during non-recording time slots, so that logger can sample data over a longer time.

## 5. Results

**Figure 14:**
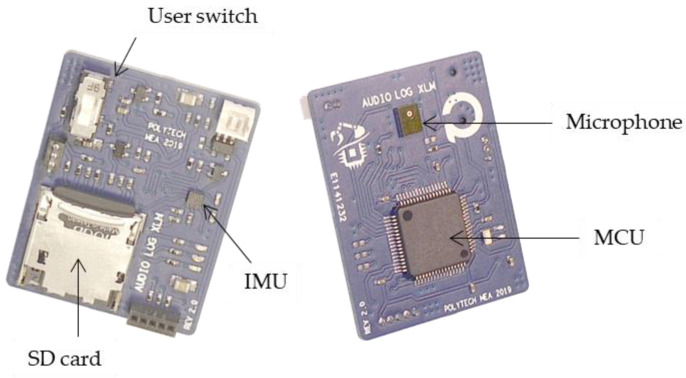
Picture of the batch-fabricated logger board.

A first production batch of 100 loggers has been completed. Figure 14 shows the electronic board with its main components. The board is 3.5 × 2.7 cm in size and weighs 4.9 g.

### 5.1. Audio Performances

Audio performance analysis are carried out using dedicated high-end audio equipment (Audio Precision APX525) to generate jitter-free clock and pure test tone and to capture both analog and PDM streams.

**Figure 15:**
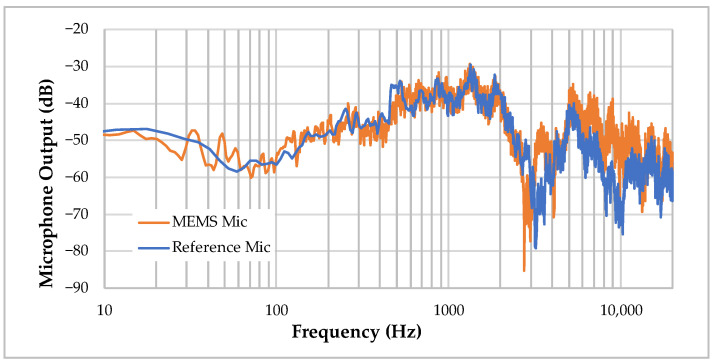
Frequency response comparison of the logger MEMS microphone with a reference microphone.

The first matter of interest in our application is the MEMS microphone frequency response. For this measure, a reference microphone with calibrated flat frequency response in the audio 20 Hz–20 kHz band is used (Earthworks, Milford, CT, USA^®^ M23) besides the MEMS under test. Both microphones are exposed to the same white noise, generated by the audio analyser, and are played by a conventional (i.e., non-flat) loudspeaker, in a standard (i.e., non-acoustically controlled) office room. The digital MEMS microphone receives its power supply and clock directly from the audio analyser, which also processes microphone output. The spectrums obtained are reported in Figure 15. The non-flat nature of the two frequency responses is likely due to (i) loudspeaker nonlinearities and (ii) room effects, so that only the difference between spectrums is relevant here. Result reveals that the MEMS microphone exhibits an acceptable frequency response with no significant localized peaks or dips. Note that spectrums have been vertically aligned in order to ease comparison, and that nothing can be concluded regarding respective SNR in this experiment.

The second concern is the effects of the embedded processing on the audio quality, in terms of signal-over-noise (SNR) ratio. For this study, the analyser provides the MEMS microphone with a steady clock and a 90 dB 1 kHz test tone at 10 cm, close to the microphone saturation level. Both the clock and microphone output PDM are then captured and are post-processed using a Scilab^®^ script that perfectly mimics the embedded digital processing within the DFSDM peripheral, the ADPCM compression, and then computes the corresponding FFT.

According to Equations (1)–(4), audio bandwidth and dynamic range depend on PDM demodulation parameters *FOSR* (over-sampling ratio) and *X* (decimation filter order). To these parameters, one should add the destructive effect of ADPCM compression, which also depends on the audio data rate, with lower degradation at higher rates.

**Figure 16:**
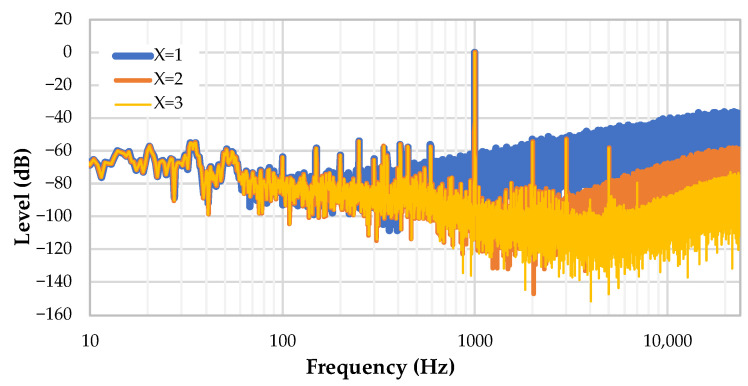
Effect of the filter order on the noise shaping.

Figure 16 shows the effect of the filter order *X* with a fixed oversampling ratio *FOSR* = 32. As expected, higher filter orders provide better smoothing and less quantization noise. However, increasing *X* also increases the dynamic range of the filter output, which we need to keep within a 16 bit data. Experimentally, with our microphone, setting *X* = 3 provided an acceptable dynamic range with no saturation when exposed to loud audio sources.

**Figure 17:**
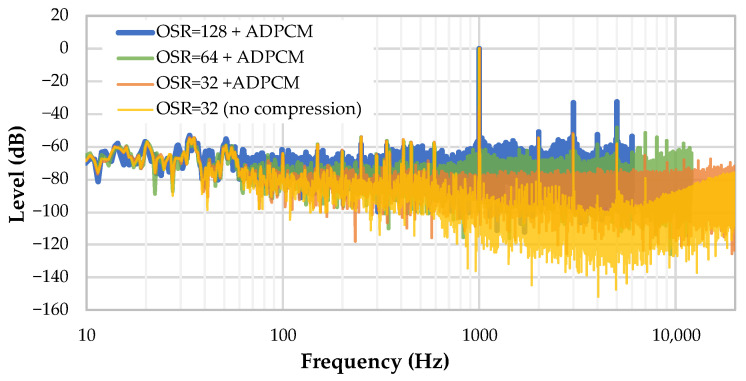
Effect of ADPCM compression on the noise floor, for various values of OSR.

Figure 17 shows the effect of ADPCM compression on the noise floor obtained with various sample rates (i.e., *FOSR* values). There is an obvious rise in the noise power as sample rate diminishes (i.e., higher values of *FOSR*). Still, the noise floor is kept around −60 dB so that no dramatic degradation is observed when compared to intrinsic microphone SNR, even at lower sample rates.

### 5.2. Recorded Data Alignment

**Figure 18:**
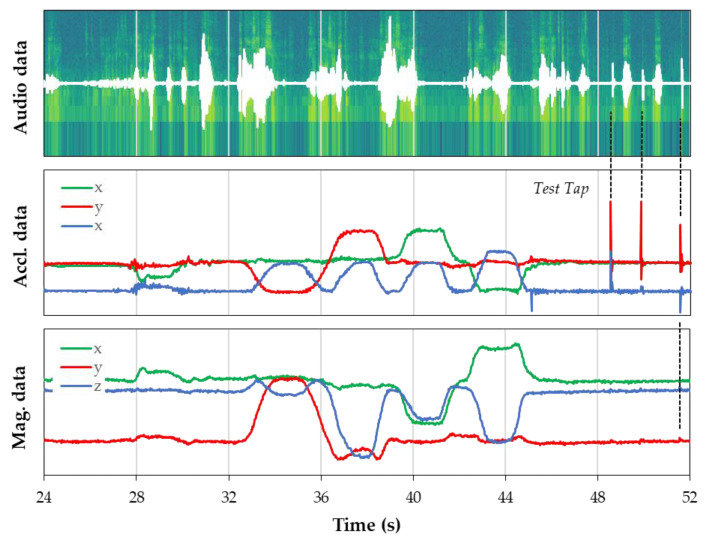
Example of time-aligned recorded data.

An example of post-processed recorded data is provided in Figure 18. As expected, audio and inertial data time alignments are perfect due to the sample-accurate timestamping approach. Visually, this is shown using gentle tapping on hard surfaces during the record session so that sharp and easy-to-locate events are produced both on the audio and inertial data. This alignment is precious considering that data analysis and classification are to be automated at some point, and that training of machine learning models will greatly benefit from such a synchronization of various information.

### 5.3. Battery Autonomy

**Figure 19:**
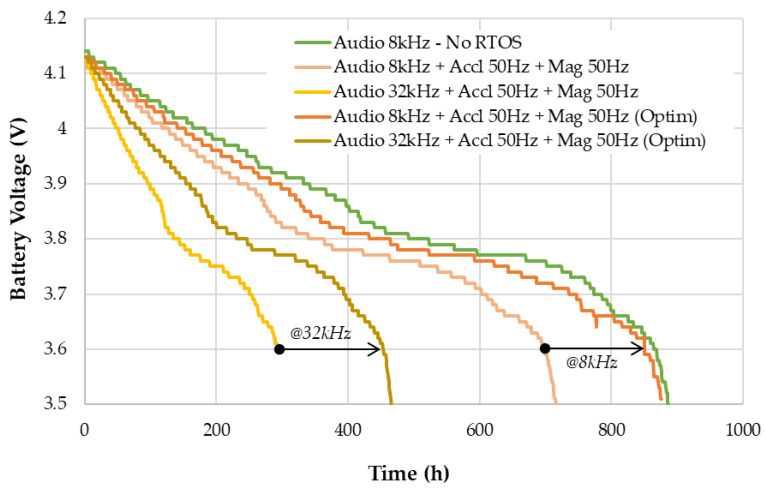
Battery voltage level over time during continuous recording (logger autonomy).

Real conditions autonomy has been measured using an 1800 mA·h Li-ion battery, with both non-optimized and optimized firmware. The logger was kept static and at room temperature for the whole duration of the experiment. Measurements are performed hourly using available MCU’s ADC and recorded onto the SD card as part of the system log. Figure 19 reports the maximum measured battery voltage over a 24 h running window. Optimized versions of the firmware exhibit a significantly extended autonomy. The 8 kHz version gets very close to an extra reference design featuring audio only, no RTOS and a straightforward sleeping scheme.

At 8 kHz audio, the logger produces nearly 900 h of continuous audio and inertial data which correspond to 15 GB of data. At 32 kHz audio, the logger still manages to record about 500 h of data for a total storage usage of 29 GB.

### 5.4. Power Contributors

**Figure 20:**
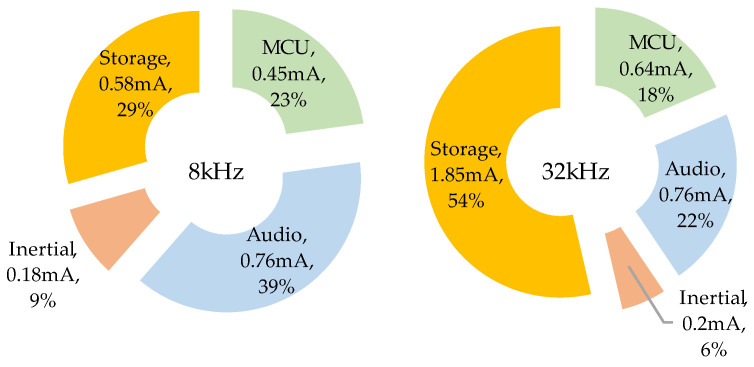
Consumption distribution among logger components.

Figure 20 presents the relative contributions of main logger tasks in the global energy consumption. These are expressed as an equivalent static current, calculated by averaging transient currents over a long period. The “Storage” part includes all SD card activity for both audio and inertial data. The “Inertial” part represents the cost of powering the sensor and collecting its data. The “Audio” part includes both the microphone supply and MCU hardware processing chain. Finally, the “MCU” part mostly concerns ADPCM compression when active, or the sleep state.

When sampling audio at 8 kHz, we observe that storage contribution is only 29% of the total energy. Audio and inertial sensors already make half of this total energy, leaving little room for improvement on the sole software side. At 32 kHz, the storage part grows up to 54%, making it the main power consumption contributor. In this case, working with a more aggressive compression algorithm (at the cost of more MCU power) might provide some improvements.

### 5.5. Related Works

Other audio loggers have been developed and are available either as commercial products or academic open-source solutions. The more direct comparison we can make of our design is with the Audiomoth [16], a former academic development now sold under the Open Acoustic Devices brand. It is an audio only recorder, with similar architecture as the one herein presented, including a MEMS analog microphone which has been successfully involved in the recent application of passive acoustic monitoring [17,18,19]. SOLO is an open-source architecture based on low-cost embedded computer [11]. As a more generic approach to recording, it is highly configurable and is an appealing solution for researcher [20,21,22], but its power consumption is rather high. On a more high-end level, both *Wildlife Acoustics* with their *Song-Meter* product range [23], and *Frontier Labs* with the *BAR-LT* device [24] make high-quality field audio recording solutions available. Despite a significantly higher cost of those devices, partly due to the quality housing, it is a relevant alternative for field recording seeing consistent usage [25,26]. It is difficult to make precise comparisons of these devices regarding power consumption because details are not always available, and requirements in terms of audio quality and features are very application specific. Nevertheless, considering available data, Table 6 provides hints regarding the current consumption of our solution with the above-listed alternatives. Power consumption also depends on SD cards choice, which is left to the end user. Based on these numbers, we see that our device is at least six times better than the closest alternative, with the added value of capturing movement data as well.

## 6. Conclusions

This paper details the design of a biologging device, covering both hardware and software dimensions. A particular focus is brought to power consumption, which is a major concern, particularly with animal-borne trackers where the autonomy/weight ratio is critical. Power savings are addressed at hardware level by a careful selection of power-efficient components, and then at software level by (i) reducing the CPU load, (ii) using audio compression to reduce storage energy, and (iii) implementing RTOS low-power strategies. The result is a logger that provides 900 h of continuous audio/inertial data recording on its 8 kHz audio configuration, which establishes the state of the art.

There is always room for improvement. For instance, investigating audio compression algorithms seeking a more efficient approach might be of interest. In addition, the use of Adaptive Frequency Scaling (AFS) [27,28] which aims at dynamically setting CPU clock frequency to its most efficient spot at any given time is yet to be investigated. Finally, on-board data filtering to reduce storage activity has shown promising results [29].

Although this paper focuses on design, let us mention that loggers have been fabricated and deployed on several species including zebras, lions, hyenas, elephants, and horses. A sealed 3D-printed enclosure has been designed for this purpose which allows for easy attachment of the logger onto existing collars. It includes a SAFT LS17500 Li-Ion 3.6 V 3.4 Ah battery weighing 23.5 g. The total logger weight is then about 60 g. Successful recordings were achieved, yet a critical issue remains regarding waterproofing. A Gore-Tex^®^ thin membrane was used to protect the microphone while being transparent regarding sound filtering. This design offers sufficient sealing to resist rain and splashes but hardly survives full immersion.

## Figures and Tables

**Table 1:** Logger DFSDM configurations summary for both the high-quality (32 kHz) and extended autonomy (8 kHz) versions of the audio front-end.

Version Name	f_clk_	*FOSR*	*IOSR*	Actual *SR*	Audio *BW*
8 kHz	2 MHz	32	8	7812.5 Hz	3.9 kHz
32 kHz		64	1	31,250 Hz	15.6 kHz

**Table 2:** Energy required for a 2 s recording without and with embedded ADPCM compression.

Vsupply = 4.2 V	I (mA)	t (ms)	×	E (mJ)
**Raw data**				
Sleep	1.35	455.10	4	9.83
Process (roundoff)	1.68	12.02	4	0.32
Store	23.34	23.47	4	8.79
**Total**				**18.94**
**ADPCM**				
Sleep	1.35	52.77	30	8.55
Process (encode)	1.68	11.40	30	2.30
Store	23.34	23.47	1	1.19
**Total**				**13.04**

**Table 3:** RAM usage.

Data Buffers	Size	73%
MIC Buffer	39.5 kB	30.8%
ADPCM Buffer	30 kB	23.4%
ACCL Buffer	12 kB	9.4%
MAG Buffer	12 kB	9.4%
Firmware		11%
RTOS Heap	11 kB	8.6%
Files management	3.7 kB	2.9%
Miscellaneous	2.4 kB	1.87%
Free	17.4 kB	16%

**Table 4:** Average supply current (mA) measured during Capture and Process states for various tick rates and low-power idle strategies.

Low-Power Mode	Tick Rate	Capture	Process	Average
None	10 Hz	-	1.6403	1.6403
None	1 kHz	-	1.6355	1.6403
None	10 kHz	-	1.6678	1.6403
Idle Hook	10 Hz	1.3210	1.6630	1.4855
Idle Hook	1 kHz	1.3301	1.6512	1.4907
Idle Hook	10 kHz	1.4733	1.6412	1.5986
Tickless Idle	10 Hz	1.3133	1.6567	1.4883
Tickless Idle	1 kHz	1.3247	1.6547	1.4905
Tickless Idle	10 kHz	1.3468	1.6350	1.5652

**Table 5:** Average supply current (mA) measured during Capture and Process states for various tick rates and low-power idle strategies.

Optimization Level (gcc)	Capture (ms)	Process (ms)	Duty-Cycle	Supply Current (mA)
−O0	322	325	50.2	1.49
−O1	527	120	18.5	1.38
−O2	532	116	17.9	1.38
−O3	531	116	17.9	1.38
−Ofast	531	115	17.9	1.38

**Table 6:** Average power consumption of commonly deployed audio recording tools.

	Sampling Rate	Supply Voltage	Supply Current	Power
**This Work**	8 kHz	3.8 V 1SLi-Ion	1.97 mA	7.5 mW
	32 kHz		3.45 mA	13.1 mW
**AudioMoth** [16]	8 kHz	4.5 V (3 × AA) or 6 V	10 mA	45 mW
	32 kHz		13 mA	58 mW
**SOLO** [11]	16 kHz	5 V	-	350 mW
**Song Meter** **Micro** [23]	8 kHz	4.5 V (3 × AA)	-	63 mW
	32 kHz			88 mW
**BAR-LT** [24]	16 kHz	3.8 V 1S Li-Ion	20.6 mA	78 mW
	32 kHz		22.6 mA	86 mW

## Data Availability

Not applicable.

## References

[1] Tuomainen U., Candolin U. (2011). Behavioural responses to human-induced environmental change. Biol. Rev..

[2] Kays R., Crofoot M.C., Jetz W., Wikelski M. (2015). Terrestrial animal tracking as an eye on life and planet. Science.

[3] Williams H.J., Taylor L.A., Benhamou S., Bijleveld A.I., Clay T.A., de Grissac S., Demšar U., English H.M., Franconi N., Gómez-Laich A. (2020). Optimizing the use of biologgers for movement ecology research. J. Anim. Ecol..

[4] Suraci J.P., Smith J.A., Chamaillé-Jammes S., Gaynor K.M., Jones M., Luttbeg B., Ritchie E.G., Sheriff M.J., Sih A. (2022). Beyond spatial overlap: Harnessing new technologies to resolve the complexities of predator–prey interactions. Oikos.

[5] Wilmers C.C., Nickel B., Bryce C.M., Smith J.A., Wheat R.E., Yovovich V. (2015). The golden age of bio-logging: How animal-borne sensors are advancing the frontiers of ecology. Ecology.

[6] Whitford M., Klimley A.P. (2019). An overview of behavioral, physiological, and environmental sensors used in animal biotelemetry and biologging studies. Anim. Biotelemetry.

[7] Lynch E., Angeloni L., Fristrup K., Joyce D., Wittemyer G. (2013). The use of on-animal acoustical recording devices for studying animal behavior. Ecol. Evol..

[8] Stidsholt L., Johnson M., Beedholm K., Jakobsen L., Kugler K., Brinkløv S., Salles A., Moss C.F., Madsen P.T. (2019). A 2.6-g sound and movement tag for studying the acoustic scene and kinematics of echolocating bats. Methods Ecol. Evol..

[9] Hill A.P., Prince P., Piña Covarrubias E., Doncaster C.P., Snaddon J.L., Rogers A. (2018). AudioMoth: Evaluation of a smart open acoustic device for monitoring biodiversity and the environment. Methods Ecol. Evol..

[10] Wijers M., Trethowan P., Markham A., Du Preez B., Chamaillé-Jammes S., Loveridge A., Macdonald D. (2018). Listening to lions: Animal-borne acoustic sensors improve bio-logger calibration and behaviour classification performance. Front. Ecol. Evol..

[11] Whytock R.C., Christie J. (2017). Solo: An open source, customizable and inexpensive audio recorder for bioacoustic research. Methods Ecol. Evol..

[12] Latorre L., Chamaillé-Jammes S. (2020). Low-Power Embedded Audio Recording using MEMS Microphones. Symp. Des. Test Integr. Packag. MEMS MOEMS.

[13] Latorre L., Miquel J., Chamaillé-Jammes S. (2021). MEMS based Low-Power Multi-Sensors device for Bio-Logging Applications. Symp. Des. Test Integr. Packag. MEMS MOEMS.

[14] Benini L., Bogliolo A., De Micheli G. (2000). A survey of design techniques for system-level dynamic power management. IEEE Trans. Very Large Scale Integr. Syst..

[15] Rodriguez-Zurrunero R., Araujo A., Lowery M.M. (2021). Methods for Lowering the Power Consumption of OS-Based Adaptive Deep Brain Stimulation Controllers. Sensors.

[16] Hill A.P., Prince P., Snaddon J.L., Doncaster C.P., Rogers A. (2019). AudioMoth: A low-cost acoustic device for monitoring biodiversity and the environment. HardwareX.

[17] Barber-Meyer S.M., Palacios V., Marti-Domken B., Schmidt L.J. (2020). Testing a New Passive Acoustic Recording Unit to Monitor Wolves. Wildl. Soc. Bull..

[18] Montgomery G.A., Belitz M.W., Guralnick R.P., Tingley M.W. (2021). Standards and Best Practices for Monitoring and Benchmarking Insects. Front. Ecol. Evol..

[19] Lapp S., Wu T., Richards-Zawacki C., Voyles J., Rodriguez K.M., Shamon H., Kitzes J. (2021). Automated detection of frog calls and choruses by pulse repetition rate. Conserv. Biol..

[20] Bradfer-Lawrence T., Gardner N., Bunnefeld L., Bunnefeld N., Willis S.G., Dent D.H. (2019). Guidelines for the use of acoustic indices in environmental research. Methods Ecol. Evol..

[21] Beason R.D., Riesch R., Koricheva J. (2019). AURITA: An affordable, autonomous recording device for acoustic monitoring of audible and ultrasonic frequencies. Bioacoustics.

[22] Darras K.F.A., Deppe F., Fabian Y., Kartono A.P., Angulo A., Kolbrek B., Mulyani Y.A., Prawiradilaga D.M. (2020). High microphone signal-to-noise ratio enhances acoustic sampling of wildlife. PeerJ.

[23] https://www.wildlifeacoustics.com/products/song-meter-micro.

[24] https://www.frontierlabs.com.au/bar-lt.

[25] Massa B., Cusimano C.A., Fontana P., Brizio C. (2022). New Unexpected Species of Acheta (Orthoptera, Gryllidae) from the Italian Volcanic Island of Pantelleria. Diversity.

[26] Vella K., Capel T., Gonzalez A., Truskinger A., Fuller S., Roe P. (2022). Key Issues for Realizing Open Ecoacoustic Monitoring in Australia. Front. Ecol. Evol..

[27] Rodriguez-Zurrunero R., Araujo A. (2021). Adaptive frequency scaling strategy to improve energy efficiency in a tick-less Operating System for resource-constrained embedded devices. Future Gener. Comput. Syst..

[28] Chen Y.L., Chang M.F., Yu C.W., Chen X.Z., Liang W.Y. (2018). Learning-Directed Dynamic Voltage and Frequency Scaling Scheme with Adjustable Performance for Single-Core and Multi-Core Embedded and Mobile Systems. Sensors.

[29] Prince P., Hill A., Piña Covarrubias E., Doncaster P., Snaddon J.L., Rogers A. (2019). Deploying Acoustic Detection Algorithms on Low-Cost, Open-Source Acoustic Sensors for Environmental Monitoring. Sensors.

